# Identification of TNM stage-specific genes in lung adenocarcinoma by genome-wide expression profiling

**DOI:** 10.3892/ol.2013.1469

**Published:** 2013-07-15

**Authors:** MING LIU, HONG PAN, FENG ZHANG, YONGBIAO ZHANG, YANG ZHANG, HAN XIA, JING ZHU, WEILING FU, XIAOLI ZHANG

**Affiliations:** 1 Department of Cardiothoracic Surgery, Affiliated Daping Hospital, Third Military Medical University, Chongqing 400042, P.R. China; 2 Department of Laboratory Diagnostics, Affiliated South-West Hospital, Third Military Medical University, Chongqing 400038, P.R. China; 3 Beijing Institute of Genomics of the Chinese Academy of Sciences, Beijing Genomics Institute, Beijing Proteomics Institute, Beijing 100029, P.R. China; 4 Department of Laboratory Diagnostics, The First Hospital Affiliated to the Chinese PLA General Hospital, Beijing 100048, P.R. China

**Keywords:** lung adenocarcinoma, tumor-node-metastasis stage, gene expression profile

## Abstract

The present study aimed to investigate the molecular basis of lung cancer development using a microarray to identify the differentially-expressed genes associated with the various tumor-node-metastasis (TNM) stages of lung adenocarcinoma. This subtype of lung cancer has increased in incidence within recent years in China. A 35K oligo gene array covering ~25,100 genes was used to screen the differentially-expressed genes among 90 lung adenocarcinoma samples of various TNM stages. To verify the data from the gene arrays, three genes [human zinc finger-containing, Miz1, PIAS-like protein on chromosome 7 (Zimp7), GINS complex subunit 2 (GINS2) and NSAID activated gene 1 (NAG-1)] were validated using quantitative (q)PCR in an alternative set of samples to the gene array. A total of 640 genes were identified that were differentially-expressed in lung adenocarcinoma compared with the surrounding normal lung tissues. From these 640 candidate genes, 10 were observed to be differentially-expressed among TNM stages I, II and IIIA, of which, the Zimp7, GINS2 and NAG-1 genes were reported for the first time to be expressed at high levels in lung adenocarcinoma. The results of the qPCR for the three genes were consistent with those from the gene array. In total, 10 candidate genes were identified to be associated with the various TNM stages of lung adenocarcinoma in the population studied, which may provide new insights into the molecular basis underlying the development of lung adenocarcinoma and offer new targets for the diagnosis, therapy and prognosis.

## Introduction

Lung cancer is one of the most common malignancies, with a high incidence and mortality rate worldwide. The 5-year survival rate is 15% for Americans, 10% for Europeans and 8.9% in developing countries. In China, there are ~400,000 new cases and 360,000 fatalities from lung cancer per year, accounting for one-third of the overall incidence of this cancer worldwide (1). Although certain mechanisms have been identified to underlie the development of lung cancer, current research is insufficient with regard to what is required to significantly improve the current diagnosis and treatment practices. Therefore, it is crucial that additional lung cancer-related genes are identified to provide new markers or targets for the diagnosis, treatment and prognosis of this disease.

The use of gene arrays, as powerful tools for the gene expression profile analysis of the whole genome, is a preferred strategy for the identification of differentially-expressed genes in cancer. They have been widely used to identify tumor-associated genes in various types of cancer, including lung carcinomas. However, the majority of gene array-based expression studies of lung cancer have ignored the differences between the clinical features of patients, including the tumor-node-metastasis (TNM) stage (2,3). The identification of differentially-expressed genes in lung cancers of various clinical statuses may provide new opportunities to improve the diagnosis and treatment of this disease.

The main histological type of lung cancer is non-small cell lung carcinoma (NSCLC), which consists of squamous cell carcinoma, adenocarcinoma and large cell lung cancer. Based on clinical practice and the published data in China, lung adenocarcinoma is an aggressive paradigm with a high chance of relapse and early metastasis. The condition has become one of the most common subtypes of lung cancer in the country, accounting for 30–40% of the overall cases.

In the present study, a 35K oligo gene array was used to identify the differentially-expressed genes that were associated with the various TNM stages of lung adenocarcinomas. The results may provide new information to decode the molecular pathogenesis of lung cancer and offer new targets for the diagnosis, treatment and prognosis of this disease.

## Materials and methods

### Specimens

Lung adenocarcinoma specimens were collected from 240 patients who underwent thoracic surgery at Southwest Hospital. All patients were primary cases with no prior history of other malignancies, and no patient had been administered chemo- or radiotherapy. In addition to the cancer tissue, the surrounding normal lung tissue was collected to be used as a control. A 1.0-cm^3^ volume of tissue was further cut into 0.5-cm^3^ blocks and placed into 5-fold volumes of RNAlater. Subsequent to being frozen for 30 min in liquid nitrogen, the tissues were kept at −80°C for later use. A section of each cancer tissue was prepared for routine hematoxylin and eosin staining to confirm the percentage of cancer cells. Only the samples in which the proportion of cancer cells was >80% were used to prepare the RNA. The specimens were reviewed pathologically to confirm the diagnosis of lung adenocarcinoma. A total of 90 samples (30 each for stages I, II and IIIA) were used for the gene array, and 150 samples (50 each for stages I, II and IIIA) were used for the validation by quantitative (q)PCR. Approval for the present study was obtained from the ethics committee of the Affiliated South-West Hospital (Chonqing, China). The study conforms to the provisions of the Declaration of Helsinki in 1995 (as revised in Tokyo 2004) and informed consent was obtained from all patients.

### Preparation of total RNA

The total RNA from each tissue sample was extracted using a Tripure™ Isolation Reagent kit (Ambion Inc., Foster City, CA, USA) according to the manufacturer's instructions. The RNA samples were purified with a Nucleospin RNA clean-up kit (Machery-Nagel Inc., Bethlehem, PA, USA), according to the manufacturer's instructions. The concentration and purity of the RNA samples were assayed with a Nanodrop spectrophotometer (Thermo Fisher Scientific Inc., Waltham, MA, USA). The integrity of the RNA was confirmed using electrophoresis in agarose gel containing formaldehyde.

### Gene array hybridization

#### 35K oligo gene array

The Jingxin^®^ 35K oligo gene array (CapitalBio Inc., Beijing, China) involved 35,000 70-mer oligonucleotide probes (human genome-wide oligo library Version 4.0; Operon Inc., Huntsville, AL, USA), covering ~25,100 genes.

#### Fluorescence-labeling of RNA samples

The Jingxin cRNA linear amplification and labeling kit (CapitalBio) was used. The RNA was first reverse transcribed to single stranded cDNA, which was used as a template to synthesize the second cDNA strand. The second strand of cDNA was used as a template to synthesize the cRNA using T7 Enzyme Mix. The cRNA was reverse transcribed to cDNA using Random Primer. The purified cDNA was used as a template to synthesize the fluorescence-labeled cDNA chain using the Klenow enzyme. Cy5 dCTP and Cy3 dCTP were used to label the test sample (tumor or surrounding normal tissue) and the Universal Human Reference RNA, respectively. The final labeled products were purified using a PCR NucleoSpin Extract II kit (Machery-Nagel Inc.). A Nanodrop spectrophotometer was used to determine the concentration and labeling efficiency of the products. The fluorescence intensities of Cy3 and Cy5 should be comparable.

#### Hybridization

Subsequent to being mixed with a hybridization buffer, the labeled products were added to the gene arrays, which were then incubated in the CapitalBio^®^ BioMixer™ II microarray hybridizing incubator overnight at 42°C. Following hybridization, the gene arrays were washed and scanned using CapitalBio LuxScan™10K-A scanners. The data was extracted and analyzed by LuxScan 3.0, BoaoAnalyzer6_step1.pl and BoaoAnalyzer6_step2.pl software products (CapitalBio Inc.), and the fluorescence intensity was normalized prior to analysis.

#### Identification of differentially-expressed genes

The genes that were differentially-expressed between the lung adenocarcinoma and surrounding normal lung tissues, and those between the various TNM stage tumors, were analyzed using significance analysis of microarrays (SAM) software (Stanford University, Stanford, CA, USA).

#### qPCR

To verify the data from the gene array, the human zinc finger-containing, Miz1, PIAS-like protein on chromosome 7 (Zimp7), GINS complex subunit 2 (GINS2) and NSAID activated gene 1 (NAG-1) were chosen as candidates for the qPCR in an alternative set of tumor samples to the gene array assay. A total of 50 samples were used per TNM stage. The primers were designed and synthesized by Takara Inc. (Dalian, China; Table I). Glyceraldehyde 3-phosphate dehydrogenase (GAPDH) was used as the internal control (Takara Inc.). The amplification was performed according to the instructions outlined in the SYBR^®^ Premix Ex Taq™ Real Time RT-PCR kit manual (Takara Inc.). The 25 μl amplification included 1 μl cDNA, 0.5 μl each of forward and reverse primers, 12.5 μl 2X SYBR Premix, 0.5 μl ROX Reference Dye I and 10 μl dH_2_O. Each sample underwent amplification three times. The reactions were performed on Rotor gene 6300 (Corbett Life Science, Australia) and the conditions were as follows: predenaturation at 95°C for 10 sec, followed by 40 cycles at 95°C for 5 sec, 52.5°C (Zimp7), 59°C (GINS2) and 62°C (NAG-1) for 15 sec and finally, 72°C for 20 sec. The relative expression was quantified using the 2(^−ΔΔCt^) method (4,5).

#### Statistical analysis

The numbers of Zimp7, GINS2 and NAG-1 transcripts between the tumor samples at the various TNM stages were compared using the Fisher's least significant difference (LSD) test using SPSS software version 18.0 (SPSS, Inc., Chicago, IL, USA). P<0.05 was considered to indicate a statistically significant difference.

## Results

### Screening the differentially-expressed genes

A two-class unpaired method from the SAM software was adopted to screen the differentially-expressed genes between 90 tumor samples of lung adenocarcinoma and their surrounding normal lung tissues. The criteria to define the difference were as follows: i) q-value of <5%; and ii) the ratio of the fluorescence intensity of Cy5 to Cy3 (i.e. fold change) as ≥2 or ≤0.5. Overall, a total of 640 candidate genes were identified to be differentially-expressed in the tumor tissues compared with the normal tissues, among which, 289 genes were upregulated and 351 were downregulated. A multiclass method from the SAM software was used to identify the differentially-expressed genes among the tumor samples at the various TNM stages (stages I, II and IIIA) from the 640 candidates with criteria of: i) q-value of <15%; and ii) fold change of ≥2 or ≤0.5. Finally, 10 genes were obtained (Table II), among which, four genes, dual-specificity mitogen activated protein (MAP) kinase phosphatase 1 (DUSP1), Zimp7, EST NP_937824 and XM_498632, were expressed at a higher level in the stage I adenocarcinoma samples than in those of stages II and IIIA. A further two genes showed a higher expression in the stage II samples than in those of stages I and IIIA (GINS2 and lymphocyte antigen 6 complex, locus K, LY6K), and four genes exhibited higher expression in the stage II and IIIA samples than in those of stage I [NAG-1, melanoma-associated antigen 3 (MAGE-A3), metastasis associated lung adenocarcinoma transcript-1 (MALAT-1) and matrix metalloproteinase 12 (MMP-12)].

### Confirming the expression of certain candidate genes by qPCR

Using GAPDH as an internal control, the ratio of the candidate gene transcript between the tumor and the matched normal tissues [tumor/normal (T/N)] was calculated. If the ratio equaled more than two, the target gene was considered to be upregulated in the tested tumor sample compared with its matched normal sample. The results showed that the Zimp7, GINS2 and NAG-1 transcripts were upregulated in lung adenocarcinoma and that the mean ratio (T/N; mean ± SD) of the three genes in 150 matched tumor and normal samples were 3.86±2.09, 6.80±4.55 and 10.80±5.61, respectively, with an upregulation positive percentage of 85.33% (128/150), 88.67% (133/150) and 96.67% (145/150), respectively. The results of the qPCR were consistent with those from the gene array.

**Figure 1:**
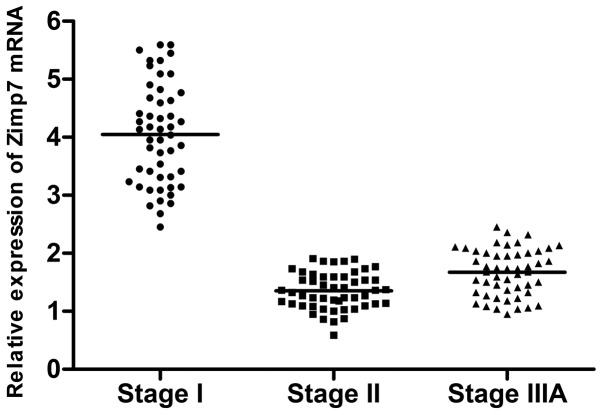
Determination of Zimp7 mRNA levels in 150 patients with lung adenocarcinoma of various TNM stages by qPCR. Data was calculated using the 2(^−ΔΔCt^) formula and analyzed with the Fisher's LSD test. Horizontal bars indicate median values. Compared with stage II and IIIA samples, Zimp7 was expressed at a significantly higher level in stage I lung adenocarcinoma (P<0.01). Zimp7, human zinc finger-containing, Miz1, PIAS-like protein on chromosome 7; TNM, tumor-node-metastasis; qPCR, quantitative PCR; LSD, least significant difference.

**Figure 2:**
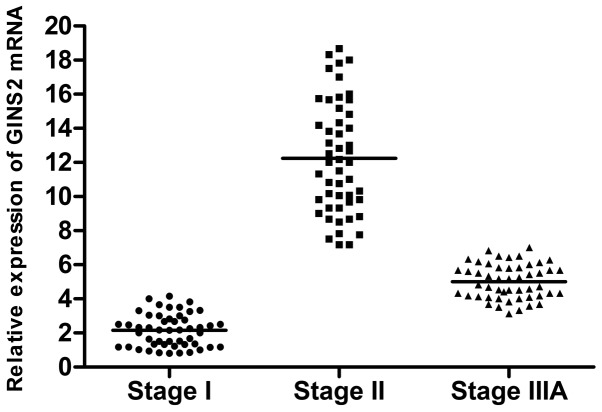
Determination of GINS2 mRNA level in 150 patients with lung adenocarcinoma of various TNM stages by qPCR. Data was calculated using the 2(^−ΔΔCt^) formula and analyzed with the Fisher's LSD test. Horizontal bars indicate median values. Compared with stage I and IIIA samples, GINS2 was expressed at a significantly higher level in stage II lung adenocarcinoma (P<0.01). GINS2, GINS complex subunit 2; TNM, tumor-node-metastasis; qPCR, quantitative PCR; LSD, least significant difference.

**Figure 3:**
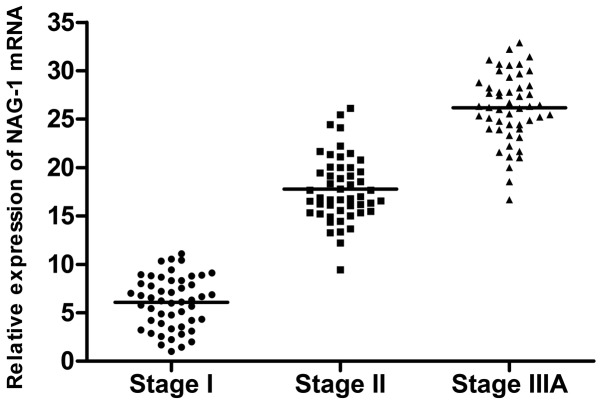
Determination of NAG-1 mRNA levels in 150 patients with lung adenocarcinoma of various TNM stages by qPCR. Data was calculated using the 2(^−ΔΔCt^) formula and analyzed with the Fisher's LSD test. Horizontal bars indicate median values. Compared with stage I samples, NAG-1 expression was significantly increased in stage II and IIIA lung adenocarcinoma (P<0.01). NAG-1, NSAID activated gene 1; TNM, tumor-node-metastasis; qPCR, quantitative PCR; LSD, least significant difference.

The relative expression levels of Zimp7 mRNA in the lung adenocarcinoma patients of stages I, II and IIIA were 4.04±0.86, 1.35±0.32 and 1.67±0.40, respectively, and for the GINS2 mRNA, the levels were 2.15±0.95, 12.23±3.27 and 5.01±1.02, respectively. The mRNA levels of NAG-1 were 6.08±2.67, 17.77±3.40 and 26.16±3.55, respectively (Figs. 1–3). Zimp7 showed a significantly higher expression level in the stage I samples than in those of stages II and IIIA, while GINS2 showed a higher level in stage II samples than in those of stages I and IIIA. NAG-1 expression was significantly increased in stages II and IIIA compared with stage I. These results were consistent with the gene array.

## Discussion

The present study identified 10 differentially-expressed genes among 90 lung adenocarcinoma tumor samples of various TNM stages (30 cases per stage) using a 35K oligo gene array. Of the 10 candidates, DUSP1 was downregulated in lung adenocarcinoma and the other nine were upregulated.

Compared with the stage II and stage IIIA samples, the stage I lung adenocarcinoma samples were marked by four differentially-expressed genes, DUSP1, Zimp7, EST NP_937824 and XM_498632.

DUSP1, also known as MKP1, negatively regulates the activity of extracellular regulated kinases (ERKs) and MAP kinases (MAPKs). MKP1 expression has been shown to progressively decrease with the development of epithelial carcinomas (6), and is one of the 20 most significantly downregulated genes in colorectal cancers (7). Studies indicate that the induction of MKP1 may significantly suppress the proliferative and metastatic abilities of NSCLC *in vitro* and *in vivo*. Therefore, MKP1 may be considered to be a potential therapeutic target in NSCLC therapy (8). In the present study, DUSP1 mRNA was significantly downregulated in the lung adenocarcinoma tissues, and the levels declined progressively with the development of this cancer, which was consistent with the results reported with regard to epithelial carcinomas. The analysis of DUSP1 expression may be useful to evaluate the disease extent and prognosis of lung adenocarcinoma.

Zimp7, also named Zmiz2, is a novel PIAS-like protein that functions as a transcriptional co-activator. Transient transfection into prostate epithelial cells showed that human Zimp7 augments the transcriptional activity of the androgen receptor (AR), which is known to be of importance in the survival of prostate cancer cells (9). The present study was the first to report the higher expression of Zimp7 mRNA in stage I lung adenocarcinomas. Whether the consequence of its high expression is also to augment the nuclear hormone receptor-regulated transcription similar to that found in prostate cancer requires further study.

Furthermore, two more genes were identified bearing unknown functions, which were expressed at higher levels in the stage I lung adenocarcinomas (EST NP_937824 and XM_498632). The potential value of these genes in the early diagnosis of lung adenocarcinoma required further evaluation later in the study.

Compared with the stage I and IIIA samples, the stage II lung adenocarcinoma samples were marked by two differentially-expressed genes (GINS2 and LY6K).

GINS2 is a member of a tetrameric complex, GINS, composed of GINS1, GINS2, GINS3 and GINS4, which most likely serves as the replicative helicase. As it has been reported that DNA replication-associated proteins exhibit diverse functions in various cells, GINS components, particularly GINS2, have been suggested as possessing a function in cell division, and more precisely in the chromosome segregation of cancer cells (10). A high level of GINS2 expression has been observed among metastasizing breast tumors. Bioinformatic analyses of published gene expression and DNA copy number studies of clinical breast tumors suggested that GINS2 was associated with the aggressive characteristics of a subgroup of breast cancers *in vivo*(11). In the present study, GINS2 mRNA was observed to be significantly highly expressed in stage II lung adenocarcinoma for the first time. Together with its expression in breast cancer, GINS2 is speculated to be a potential metastasis-promoting gene, and its overexpression may participate in lung adenocarcinoma metastasis.

LY6K is a cancer testis antigen located on chromosome 8q24.3. Gene expression profile analyses of NSCLC revealed that LY6K was specifically expressed in the testis and transactivated in the majority of NSCLCs. Immunohistochemical staining confirmed that LY6K overexpression was associated with a poor prognosis for patients with NSCLC (12). Cell viability assays demonstrated that the significant inhibition of cell growth, migration and invasion occurred in LY6K-knockdown bladder cancer cell lines (13). The present data show that the LY6K transcript was significantly overexpressed in the stage II lung adenocarcinoma samples, suggesting that the upregulation of the LY6K gene may contribute to the development of this cancer, and therefore, that LY6K may potentially be used for predicting the prognosis of lung adenocarcinoma, as previously reported for NSCLC (12).

Compared with the stage I samples, NAG-1, MAGE-A3, MALAT-1 and MMP-12 were significantly upregulated in stage II and IIIA lung adenocarcinoma samples.

NAG-1 was identified as a divergent member of the TGF-β superfamily. Studies of NAG-1 expression in tumors have been inconsistent. Using immunohistochemistry, a study by Kim *et al*(14) showed that NAG-1 expression is downregulated in colon tumors. NAG-1 expression has also been shown to be absent in squamous metaplastic tracheal epithelium, whereas positive expression has been observed in the normal tracheobronchial epithelia (15). In contrast, a high expression of NAG-1 is also frequently observed in certain tumors. NAG-1 has been identified as highly expressed in melanoma, and metastatic melanoma biopsies have displayed a strong expression of MIC-1 compared with the primary melanoma biopsies (16). Brown *et al*(17) reported that NAG-1 expression was markedly upregulated in colorectal cancers and that serum NAG-1 levels were higher in patients with a higher TNM stage. The apparent dichotomy of NAG-1 expression in tumors raises the possibility that NAG-1 plays distinctly differing roles at various stages of tumor progression. The gene suppresses tumorigenesis at the early stages of cancer development and promotes tumor invasiveness and survival at more advanced stages of the disease. This is not unexpected since NAG-1 is a member of the TGF-β superfamily, but an explanation for this change in biological activity is not clearly understood at the present time. The present data first revealed that the NAG-1 transcript was upregulated in lung adenocarcinomas in contrast with data from a previous report on squamous metaplastic tracheal epithelia, in which no expression was identified. The expression level was also markedly increased with the progression of the disease, similar to that in colorectal cancer as reported by Brown *et al*(17). Thus, NAG-1 may be used as a potential marker to asses the prognosis of lung adenocarcinoma.

MAGE-A3 is a cancer testis antigen that is expressed in cancer cells, but not in normal tissues. MAGE-A3 is a promising target for anticancer immunotherapy as it is exclusively presented on the cell surface of cancer cells and may be associated with an aggressive cancer phenotype. Sienel *et al*(18) compared the expression of MAGE-A3 in stage I and II NSCLCs and observed that in comparison with stage I tissues, the rate of MAGE-A3-positive tumors was significantly increased in stage II tissues, which was in agreement with its possible role in tumor metastasis. Consistent with the literature, the expression of MAGE-A3 mRNA was significantly increased in the present study in stage II and IIIA lung adenocarcinoma tissues compared with those of stage I. MAGE-A3 may also be a candidate target for the immunotherapy of lung adenocarcinoma.

MALAT-1 is a novel non-coding RNA of >8,000 nucleotides that is expressed on chromosome 11q13. Short-interfering RNA-mediated MALAT-1 silencing has been shown to impair the *in vitro* cell motility of lung adenocarcinoma cells (19). MALAT-1 has been demonstrated to be significantly associated with metastasis in NSCLC patients, and may be used as prognosis-predicting parameter for early-stage NSCLC (20). The present study identified that the expression of MALAT-1 was significantly higher in the stage II and IIIA samples than in stage I lung adenocarcinoma, which was consistent with the published data. The present results demonstrate the significance of MALAT-1 for the prediction of the metastasis and prognosis of lung adenocarcinoma.

MMP-12, also known as macrophagic elastase, is a member of the MMP family that binds to certain substrates, including elastin, type IV collagen and fiber junction protein. Extensive studies are available with regard to the role of MMPs in the invasion and metastasis of different cancers (21). We compared the MMP-12 mRNA levels between cancer tissues and matched surrounding normal tissues, between TNM stage I and stage II/III, as well as between tumors with lymph node metastasis and without, in cases of NSCLC. The data showed that MMP-12 was present in all the tested cancer tissues, but not in the normal tissues. There was a significantly higher expression level in the stage II/III samples than in those of stage I, and a higher level in the cancers with lymph node metastasis than in those without. Consistent with published data (22), the present gene array-based study of various TNM stages of lung adenocarcinoma further confirmed the role of MMP-12 in the metastasis of NSCLC and its value as a potential marker for predicting the prognosis of the disease.

In summary, the present study presents the association between the genome-wide gene expression profiles and the various TNM stages of lung adenocarcinoma in a study population, thus providing a preliminary analysis of the TNM stage-related genes that are present in lung adenocarcinomas. The majority of the 10 genes that were identified in the present study are present in lung cancer, and the results further demonstrate their essential roles in the development of lung adenocarcinoma. However, the three remaining genes, which have not been reported previously in lung cancer, have been indicated to be involved in cancers at other levels. Further study with regard to the roles of these genes in lung cancer would be of great value.

## Figures and Tables

**Table I:** Forward and reverse primers of Zimp7, GINS2 and NAG-1 genes for qPCR amplification.

Primer	Sequence (5′→3′)
Zimp7	
Forward	CGGGTCACCATTTCCTCCAGTC
Reverse	GGGGCAACGCTCACACCAGATAC
GINS2	
Forward	CAGACGAATGGCATGGCTTTTAC
Reverse	GCGGGTGCTCTTAGGCTCTC
NAG-1	
Forward	GCCCGCCAGCTACAATCC
Reverse	GGCAGGAATCGGGTGTCTCA

Zimp7, human zinc finger-containing, Miz1, PIAS-like protein on chromosome 7; GINS2, GINS complex subunit 2; NAG-1, NSAID activated gene l; qPCR, quantitative PCR.

**Table II:** Ten differentially-expressed genes identified to be associated with various lung adenocarcinoma TNM stages.

Symbol	GB. accession	Description	Fold change	Contrast-1	Contrast-2	Contrast-3
DUSP1	NM_004417	Dual specificity MAP kinase phosphatase 1	0.41^a^	1.91	−1.50	−0.30
Zimp7	NM_031449.3	Human zinc finger-containing, Miz1, PIAS-like protein on chromosome 7	2.39^b^	2.66	−1.30	−1.01
EST	NP_937824	34 kDa protein	2.36^b^	2.16	−1.52	−0.47
EST	XM_498632	Hypothetical gene supported by BC022385; BC035868; BC048326	3.67^b^	2.10	−1.18	−0.20
GINS2	NM_016095.2	GINS complex subunit 2	5.44^b^	−2.08	2.02	0.04
LY6K	NM_017527.3	Lymphocyte antigen 6 complex, locus K	12.96^b^	−0.96	1.50	−0.40
NAG-1	NM_004864.2	NSAID activated gene 1	4.81^b^	−1.03	0.56	1.64
MAGE-A3	NM_005362.3	Melanoma-associated antigen 3	6.30^b^	−1.02	0.87	1.97
MALAT-1	NR_002819.2	Metastasis associated lung adenocarcinoma transcript 1	4.03^b^	−1.44	0.74	1.90
MMP-12	NM_002426.4	Matrix metalloproteinase-12	5.42^b^	−2.08	0.94	2.02

Fold change: tumor tissues/normal tissues, fold change ≥2.0 or ≤0.5.

a down- or

b up-regulated in lung adenocarcinoma.

Contrast-n: the significant difference between stage I, II and IIIA groups of samples analyzed by the significance analysis of microarrays (SAM) software. TNM, tumor-node-metastasis; MAP, mitogen activated protein; GB, GenBank.
